# TERT mutations correlate with higher TMB value and unique tumor microenvironment and may be a potential biomarker for anti‐CTLA4 treatment

**DOI:** 10.1002/cam4.3376

**Published:** 2020-08-18

**Authors:** Huahua Li, Jia Li, Chenyue Zhang, Chenxing Zhang, Haiyong Wang

**Affiliations:** ^1^ Department of Integrated Chinese and Western Medicine Affiliated Cancer Hospital of Zhengzhou University and Henan Cancer Hospital Zhengzhou China; ^2^ Department of Integrated Therapy Fudan University Shanghai Cancer Center Shanghai Medical College Shanghai China; ^3^ Department of Nephrology Shanghai Children's Medical Center Shanghai Jiao Tong University School of Medicine Shanghai China; ^4^ Department of Internal Medicine‐Oncology Shandong Cancer Hospital and Institute Shandong First Medical University and Shandong Academy of Medical Sciences Jinan China

**Keywords:** *anti‐CTLA4* treatment, immune cells infiltration, melanoma, *TERT* mutation, *TMB*

## Abstract

Immune checkpoint inhibitors (*ICIs*) have recently changed therapeutic paradigms for patients across multiple cancer types. However, current biomarkers cannot accurately predict responses to *ICIs*. Telomerase reverse transcriptase (*TERT*) mutations lead to an aberrant upregulation of *TERT* expression, and ultimately allow telomere maintenance, thus supporting immortalization of cancer cells. This study aimed to investigate whether the *TERT* mutation is a potential predictor of *ICI* treatment across all cancer types. *TERT* mutations positively correlated with a higher tumor mutational burden (*TMB*) value, neoantigen load, and tumor purity. Lymphocyte infiltration, macrophage regulation, interferon‐gamma (*IFN‐γ*) response, and transforming growth factor‐β (*TGF‐β*) response which was representative immune‐expression signatures, all had higher signature scores in the *TERT* mutation group. Activated *CD4 T* cell, naïve *B* cell, activated dendritic cell, *M0* macrophage, *M1* macrophage, neutrophil, resting *NK* cell, and plasma cells all had relatively higher immune scores in the *TERT* mutation group, whereas *Th* series cells, memory *B* cell, resting mast cells, monocytes, and activated *NK* cells had lower immune scores. Notably, in the subgroup analysis of monotherapy and combination *ICI* treatment, only in the anti‐cytotoxic‐T‐lymphocyte‐associated antigen 4 (*anti‐CTLA4*) group, patients with *TERT* mutations had a better prognosis, especially for melanoma. Therefore, *TERT* mutations were closely related to a higher *TMB* value and unique tumor microenvironment, which may be the reason that *TERT* mutations may be a potential biomarker for anti‐*CTLA4* treatment.

## Graphical abstract

TERT mutations were positively related with higher TMB value and lower immune cells infiltration, which may lead to an immunosuppressive microenvironment. TERT mutations may be a potential biomarker for anti‐CTLA4 treatment, but not programmed cell death‐1 (PD‐1)/programmed cell death‐L1 (PD‐L1) blockade and combination treatment across pan‐cancer types.

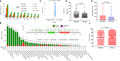

## 1 INTRODUCTION

Immune checkpoint inhibitors (*ICIs*), such as anti‐cytotoxic‐T‐lymphocyte‐associated antigen 4 (*anti‐CTLA4*) (ipilimumab), anti‐programmed cell death 1 (*anti‐PD1*) (nivolumab, pembrolizumab, and cemiplimab) and anti‐programmed cell death ligand 1 (*anti‐PD‐L1*) (atezolizumab, avelumab, and durvalumab), have recently changed the therapeutic paradigm for patients across multiple cancer types.^1^ Currently, *ICIs* have been approved by the Food and Drug Administration for the treatment of numerous cancers, because of their significant and durable clinical response in a subset of patients with certain types of cancer.^2^ However, there are also dilemmas, such as low response rates of checkpoint inhibitor monotherapy, significantly higher toxicity of combination treatment, and high treatment costs.^3^, ^4^, ^5^ Therefore, early identification of potential beneficiaries from *ICIs* would be a greatly important step forward. Ideal biomarkers would be able to stratify patients who are more sensitive to immunotherapy and monitor the treatment response in real‐time.

Programmed cell death‐L1 (*PD‐L1*) expression has been identified as one of the biomarkers used in response to *ICIs*.^6^ The percentage of *PD‐L1* expression in the tumor tissue can be used as a predictor of the efficacy of pembrolizumab in non‐small cell lung cancer (*NSCLC*).^7^ However, because of inaccurate quantification, lack of standardization across platforms, and inconsistent scoring systems, *PD‐L1* expression cannot accurately predict responses to *ICIs* in some types of cancer.^8^ The tumor mutational burden (*TMB*) has also recently gained popularity as a predictive biomarker associated with *ICI* responses. Patients with high *TMB* have obtained a higher overall response rate and median progression‐free survival, and therefore have better efficacy in immunotherapy for *NSCLC*.^9^, ^10^ Moreover, cancer *DNA* mismatch repair gene mutations could also be used as clinically applicable biomarkers.^11^, ^12^ However, the non‐uniform calculation, various sequencing approaches, and exorbitant expenses have also rendered them as not optimal indicators of *ICI* responses.^13^, ^14^ Therefore, new biomarkers are urgently needed for the identification of patients who will most likely benefit from *ICIs*, and even for treatment monitoring.

The telomerase reverse transcriptase (*TERT*) gene encodes the catalytic subunit of the telomerase complex, which maintains chromosomal ends, thus supporting the immortalization of cancer cells.^15^, ^16^ *TERT* mutations include multiple cancer‐specific genetic alterations, such as *TERT* promoter mutations,^17^, ^18^ *TERT* amplification,^19^ *TERT* rearrangements,^20^, ^21^ and *TERT* transcriptional activation.^22^ *TERT* mutations may cause aberrant up‐regulation of *TERT* expression. In addition, the increase in *TERT* expression may lead to the unlimited proliferative capacity of tumor cells, which is an important factor in tumorigenesis.^23^ *TERT* mutations are also outlined as markers of tumor aggressiveness and poor prognosis in several human cancer types.^24^, ^25^, ^26^, ^27^, ^28^ A recent study revealed that in bladder cancer, *TERT* promoter mutation appeared to be a potential predictive marker of response to Bacillus Calmette‐Guérin treatment which was regarded as one of the first and most successful oncological immunotherapy.^29^ Therefore, *TERT* mutations may be used as a predictive marker for *ICI* responses.

In this study, using public databases, we analyzed the *TERT* mutation spectrum to elucidate the correlation between *TERT* mutation and *TMB* and immune cell infiltration. Notably, *TERT* mutant patients may benefit from *anti‐CTLA4* treatment, especially for melanoma. Together, *TERT* mutation could be a promising potential prognostic biomarker for *anti‐CTLA4* responses. This may be related to the high *TMB* value and unique tumor microenvironment (*TME*).

## 2 MATERIALS AND METHODS

### 2.1 Data source and processing

All data in this study were selected from the *cBioPortal* database (https://www.cbioportal.org).^30^ The *MSK‐IMPACT* Clinical Sequencing Cohort including 10 336 patients or 10 945 samples was selected.^31^ All mutations with copy number alteration (*CNA*) data including fusion, amplification, deep deletion, and multiple alterations of *TERT* were considered. We also obtained information on the *TMB* score,^13^ neoantigen load,^32^ and tumor purity^31^ related to *TERT* mutations through public databases, eliminating information with unclear *TERT* mutation data.

In the relationship analysis between *TERT* mutation and immunotherapy, we also selected the *TMB* and immunotherapy cohort,^13^ which consisted of 1661 patients with various cancer types with available genomic, *TMB*, survival, and immunotherapy information. This study was mainly based on the public database and personal privacy information was not involved, so informed consent was not required.

### 2.2 Tumor immune estimation resource analysis

The tumor immune estimation resource (*TIMER*) algorithm database (https://cistrome.Shinyapps.io/timer/) is used to comprehensively investigate the molecular characterization of tumor–immune interactions.^33^ *TIMER* provides a module “*DiffExp*” to explore target genes expressed in tumors and adjacent normal tissues. The “*DiffExp*” module is used to study the differential expression between tumor and adjacent normal tissues for target genes across all the cancer genome atlas tumors. Distributions of gene expression levels are displayed using box plots, with statistical significance of differential expression evaluated using the Wilcoxon test.

### 2.3 Identification of neoantigens

We downloaded supplementary materials from the literature (https://www.ncbi.nlm.nih.gov/pmc/articles/PMC5982584/),^32^ and carefully analyzed information on neoantigen load related to *TERT* mutations. The specific steps for determining neoantigens load in the literature were as follows: potential neoantigenic peptides were identified using *NetMHCpan* 3.0, based on human lymphocyte antigen (*HLA*) types derived from RNA‐seq using *OptiType* (https://portal.gdc.cancer.gov/). First, all pairs of major histocompatibility complex (*MHC*) and minimal mutant peptide were input into *NetMHCpan* v3.0 by the *HLA* calls of each sample from *OptiType*. Second, peptides containing amino acid mutations were identified as potential antigens, based on predicted binding to autologous *MHC* (IC50 < 500 nmol/L) and detectable gene expression meeting an empirically determined threshold of 1.6 transcripts per million. Specifically, somatic nonsynonymous coding single nucleotide variants and indel variants were extracted from the *MC3* variant file (mc3.v0.2.8.CONTROLLED.maf) with filters.^32^, ^34^

### 2.4 Identification of the immune‐expression signature

We also applied the supplementary materials in this literature (https://www.ncbi.nlm.nih.gov/pmc/articles/PMC5982584/),^32^ and then identified the immune‐expression signature related to *TERT* mutations. The iterative binary bi‐clustering of gene sets (*iBBiG*) algorithm (http://www.bioconductor.org/packages/release/bioc/html/iBBiG.html) was used for meta‐gene set analysis of large numbers of gene expression datasets in the literature.^35^ The iterative algorithm extracted groups of phenotypes from multiple studies that were associated with similar gene sets and identified similarity blocks within the matrix of signature scores. The five identified representative signatures were as follows: lymphocyte infiltration, macrophage Regulation, interferon‐gamma (*IFN‐γ*) response, transforming growth factor‐β (*TGF‐β*) response, and wound healing.

### 2.5 Quantification of immune cell infiltration

Using supplementary materials downloaded from the literature (https://www.ncbi.nlm.nih.gov/pmc/articles/PMC5982584/),^32^ we quantified immune cell infiltration in the *TERT* mutation and wild type group. The relative fraction of immune cell types within the leukocyte compartment was estimated using *CIBERSORT* (https://cibersort.stanford.edu/). The proportion of these cells was multiplied to yield corresponding estimates in terms of the overall fraction in tissue. Moreover, the numerical values were aggregated in various combinations to produce abundant comprehensive cell categories. *CIBERSORT* uses a set of immune cell reference profiles to derive a base (signature) matrix that could be applied to mixed samples to determine the relative proportions of immune cells.^36^

### 2.6 *TISIDB* analysis

The *TISIDB* database (http://cis.hku.hk/TISIDB) integrated multiple types of data resources in oncoimmunology and reported 988 genes related to anti‐tumor immunity.^37^ *TISIDB* integrated five types of data resources to annotate each gene with 10 categories of knowledge. The “*Drug*” tab of the database enabled the analyses of the related drugs targeting the gene, which was helpful in designing a combinatory treatment with immunotherapy.

### 2.7 Statistical analysis

The Kaplan‐Meier method was used to calculate the survival probability and the log‐rank test was used to compare the survival curves. Data between the two groups were compared using the two‐tailed unpaired *t* test or Wilcoxon rank‐sum test. All reported *P* values are two‐tailed, and for all analyses, a *P* < .05 is considered statistically significant.

## 3 RESULT

### 3.1 Characteristic of *TERT* mutation spectrum and relationship between *TERT* mutation and *TMB*

**FIGURE 1:**
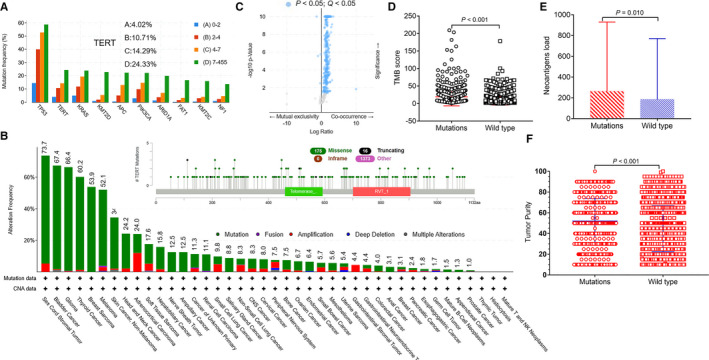
*TERT* mutation spectrum and its relationship with immunocompetence. A, Mutation frequency of the top 10 genes and mutation frequency of *TERT* (%). B, Waterfall chart of *TERT* mutation and *CNA* change and types of *TERT* mutations. C, Mutant genes that co‐occur with *TERT* mutations. The blue dots represented statistically significant co‐expressed genes (n = 1564, *P* < .05). D, Relationship between *TERT* mutation and *TMB* score (mutations = 521, wild type = 1140, 19.45 ± 26.02 vs 8.54 ± 12.71, *P* < .001) (mean ± SD). E, Relationship between *TERT* mutation and neoantigens load (mutations = 386, wild type = 6055, 264.60 ± 665.50 vs 185.70 ± 585.10, *P* = .010) (mean ± SD). F, The relationship between *TERT* mutation and tumor purity (mutations = 521, wild type = 1140, 51.92 ± 21.88 vs 44.48 ± 22.18, *P* < .001) (mean ± SD)

Tumor mutational burden is related to the number of gene mutations.^14^ To understand the relationship between gene mutations and total number of gene mutations. We divided the number of gene mutations into four groups (0‐2; 2‐4; 4‐7; 7‐455). Figure 1A demonstrates that tumor protein p53 (*TP53*), *TERT*, and *KRAS* were ranked in the top three mutations. Among them, the mutation frequencies of *TERT* in the four groups were as follows: A, 4.02%, B, 10.71%, C, 14.29%, and D, 24.33%. We further analyzed the waterfall chart of *TERT* mutation and *CNA* change. Figure 1B shows that sex cord‐stromal tumor, bladder cancer, and glioma were the top three *TERT* mutation frequencies, while thymic tumor, histiocytosis, and mature *T* and *NK* neoplasms were the last three. Figure 1C shows the mutant genes that co‐occur with *TERT* mutations in 1564 *TERT* mutation samples. Compared with *TERT* wild type, *TERT* mutations co‐mutated with most genes. The blue dots indicate statistically significant co‐expressed genes (*P* < .05).

We analyzed the relationship between *TERT* mutation and *TMB* score. The results showed that the *TMB* score of the *TERT* mutation group (n = 521) was significantly higher than that of the wild‐type group (n = 1140) (Figure 1D, 19.45 ± 26.02 vs 8.54 ± 12.71, *P* < .001) (mean ± SD). We further tested the relationship between *TERT* mutation and neoantigen load. Figure 1E shows that patients with *TERT* mutation (n = 386) had higher neoantigen load than wild‐type patients (n = 6055) (Figure 1E, 264.60 ± 665.50 vs 185.70 ± 585.10, *P* = .010) (mean ± SD). Figure 1F also demonstrated that tumor purity was higher in the *TERT* mutation group (n = 521), than in the wild type group (n = 1140) (51.92 ± 21.88 vs 44.48 ± 22.18, *P* < .001) (mean ± SD).

### 3.2 Relationship between *TERT* mutation and immune cell infiltration and its prognostic value

**FIGURE 2:**
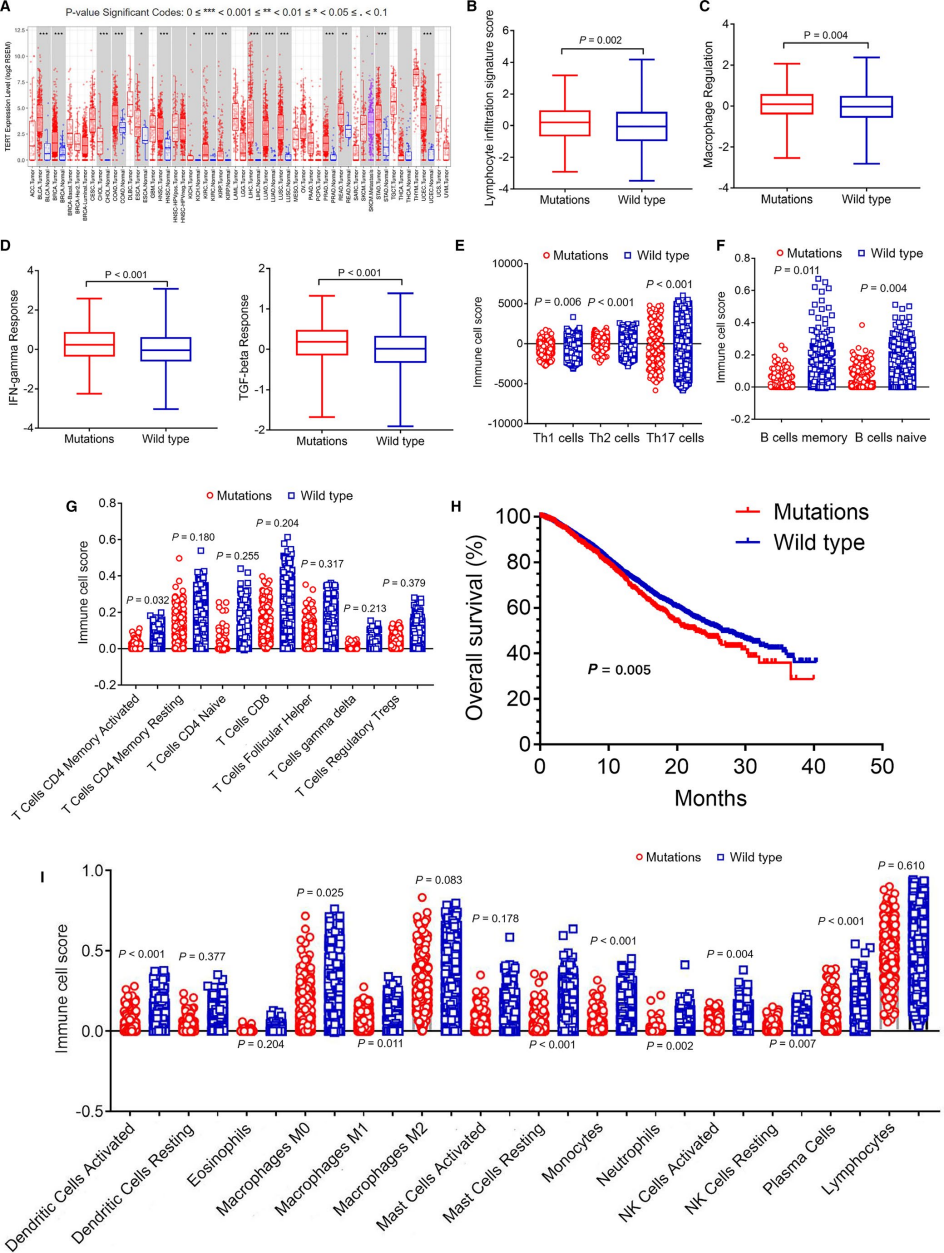
Relationship between *TERT* mutation and immune cell infiltration and its prognostic value. A, *TERT* expression of different tumor types and adjacent normal tissue in *TIMER* database. The red box plot represents tumor tissue, and the blue box plot represents adjacent normal tissue. 0≤***<0.001≤**<0.01≤*<0.05. B‐E, Relationship between *TERT* mutations and immune‐expression signatures (lymphocyte infiltration, macrophage regulation, *IFN‐γ* response, and *TGF‐β* response) (mutations = 551, wild type = 8953, 0.1386, −2.9184 to 3.1753 vs −0.0452, −3.4861 to 4.1743, *P* = .002; 0.0501, −2.5456 to 2.0633 vs −0.0448, −2.8156 to 2.3769, *P* = .004; 0.2394, −2.2508 to 2.5762 vs 0.0333, −3.0325 to 3.0649, *P* < .001; 0.1403, −1.6772 to 1.3212 vs −0.0295, −1.9033 to 1.3826, *P* < .001) (mean, minimum–maximum). The red box plot represents the *TERT* mutation group, and the blue box plot represents the *TERT* wild‐type group. F, Relationship between *TERT* mutations and Th series cell infiltration (*Th1*, *Th2*, and *Th17*) (mutations = 551, wild type = 8953, −576.1 ± 30.96 vs −485.8 ± 7.886, *P* = .006; 216.4 ± 29.89 vs −31.98 ± 8.901, *P* < .001; −667.3 ± 88.15 vs −201.6 ± 23.94, *P* < .001) (mean ± SEM). G, Relationship between *TERT* mutations and B series cell infiltration (B cells memory and B cells naïve) (mutations = 551, wild type = 8953, 0.0185 ± 0.0014 vs 0.0235 ± 0.0005, *P* = .011 and 0.0447 ± 0.0023 vs 0.0378 ± 0.0006, *P* = .004) (mean ± SEM). H, Relationship between *TERT* mutations and T series cell infiltration (only a significant difference in the amount of activated memory *CD4 T* cells, mutations = 551, wild type = 8953, 0.0070 ± 0.0007 vs 0.0054 ± 0.0002, *P* = .032) (mean ± SEM). I, Relationship between *TERT* mutations and other immune cell types. Significant differences in nine types of immune cells: activated dendritic cells (0.0232 ± 0.0018 vs 0.0134 ± 0.0003, *P* < .001) (mean ± SEM), M0 macrophages (0.0864 ± 0.0047 vs 0.0759 ± 0.0011, *P* = .025) (mean ± SEM), M1 macrophages (0.0518 ± 0.0020 vs 0.0468 ± 0.0005, *P* = .011) (mean ± SEM), resting mast cells (0.0313 ± 0.0021 vs 0.0468 ± 0.0007, *P* < .001) (mean ± SEM), monocytes (0.0300 ± 0.0016 vs 0.0384 ± 0.0005, *P* < .001) (mean ± SEM), neutrophils (0.0076 ± 0.0008 vs 0.0055 ± 0.0002, *P* = .002) (mean ± SEM), activated *NK* cells (0.0312 ± 0.0015 vs 0.0357 ± 0.0004, *P* = .004) (mean ± SEM), resting *NK* cells (0.0164 ± 0.0011 vs 0.0134 ± 0.0003, *P* = .007) (mean ± SEM), and plasma cells (0.0551 ± 0.0030 vs 0.0431 ± 0.0006, *P* < .001) (mean ± SEM). The red circle diagram represents *TERT* mutation group, and the blue block diagram represents the *TERT* wild‐type group. J, Overall survival of patients with *TERT* mutations vs wild type in the *cBioPortal* database (22.58 mo vs 26.56 mo, *P* = .005). The red curve represents the *TERT* mutation group, and the blue curve represents the *TERT* wild‐type group

To examine *TERT* expression in various tumors, we measured its expression in different types of tumors using the *TIMER* database. Figure 2A has demonstrated that *TERT* expression was higher in a variety of tumors than adjacent normal tissue. Using supplementary materials downloaded from the literature,^32^ we collected 9504 patients, including 551 patients with *TERT* mutations. We first analyzed the correlation between *TERT* mutation and immune‐expression signatures. Lymphocyte infiltration, macrophage regulation, *IFN‐γ* response, and *TGF‐β* response which were representative immune‐expression signatures, all had higher gene signature score in *TERT* mutation group (Figure 2B‐E, 0.1386, −2.9184 to 3.1753 vs −0.0452, −3.4861 to 4.1743, *P* = .002; 0.0501, −2.5456 to 2.0633 vs −0.0448, −2.8156 to 2.3769, *P* = .004; 0.2394, −2.2508 to 2.5762 vs 0.0333, −3.0325 to 3.0649, *P* < .001; 0.1403, −1.6772 to 1.3212 vs −0.0295, −1.9033 to 1.3826, *P* < .001) (mean, minimum–maximum).

We further analyzed the relationship between *TERT* mutations and immune cells. Among *Th* series cells, including *Th1*, *Th2*, and *Th17*, the immune cell score of the *TERT* mutation group was lower that of the wild‐type group (Figure 2F, −576.1 ± 30.96 vs −485.8 ± 7.886, *P* = .006; −216.4 ± 29.89 vs −31.98 ± 8.901, *P* < .001; −667.3 ± 88.15 vs −201.6 ± 23.94, *P* < .001) (mean ± SEM). It is similar, in B series cells, which contained memory and naïve B cells. As shown in Figure 2G, the immune cell score of memory B cells in the *TERT* mutation group was also lower (0.0185 ± 0.0014 vs 0.0235 ± 0.0005, *P* = .011) (mean ± SEM).The immune cell score of naïve B cells in the *TERT* mutation group was higher (0.0447 ± 0.0023 vs 0.0378 ± 0.0006, *P* = .004) (mean ± SEM). Subsequently, we analyzed the distribution of T series cells in the *TERT* mutation and wild‐type group, such as activated *CD4* memory T cells, resting *CD4* memory T cells, naïve *CD4* T cells, *CD8* T cells, follicular helper T cells, gamma delta T cells, and regulatory T cells. Only the immune score of activated *CD4* memory T cell in the *TERT* mutation group was higher than that in the wild‐type group, and there was a significant difference (Figure 2H, 0.0070 ± 0.0007 vs 0.0054 ± 0.0002, *P* = .032). Finally, we analyzed the relationship between *TERT* mutation and other immune cell types. It was found that there were differences in nine types of immune cells (Figure 2I), such as activated dendritic cells (0.0232 ± 0.0018 vs 0.0134 ± 0.0003, *P* < .001) (mean ± SEM), M0 macrophages (0.0864 ± 0.0047 vs 0.0759 ± 0.0011, *P* = .025) (mean ± SEM), M1 macrophages (0.0518 ± 0.0020 vs 0.0468 ± 0.0005, *P* = .011) (mean ± SEM), resting mast cells (0.0313 ± 0.0021 vs 0.0468 ± 0.0007, *P* < .001) (mean ± SEM), monocytes (0.0299 ± 0.0016 vs 0.0384 ± 0.0005, *P* < .001) (mean ± SEM), neutrophils (0.0076 ± 0.0008 vs 0.0055 ± 0.0002, *P* = .002) (mean ± SEM), activated NK cells (0.0312 ± 0.0015 vs 0.0357 ± 0.0004, *P* = .004) (mean ± SEM), resting NK cells (0.0164 ± 0.0011 vs 0.0134 ± 0.0003, *P* = .007) (mean ± SEM), and plasma cells (0.0551 ± 0.0030 vs 0.0431 ± 0.0006, *P* < .001) (mean ± SEM).

In analyzing the association between *TERT* mutation and overall survival (*OS*) in the cBioPortal database. Kaplan‐Meier survival analysis showed that patients with *TERT* mutation (n = 1166) showed a significantly shorter median *OS* than the wild‐type population (n = 6369) (Figure 2J, 22.58 months vs 26.56 months, *P* = .005).

### 3.3 Relationship between *TERT* mutations and ICIs

**FIGURE 3:**
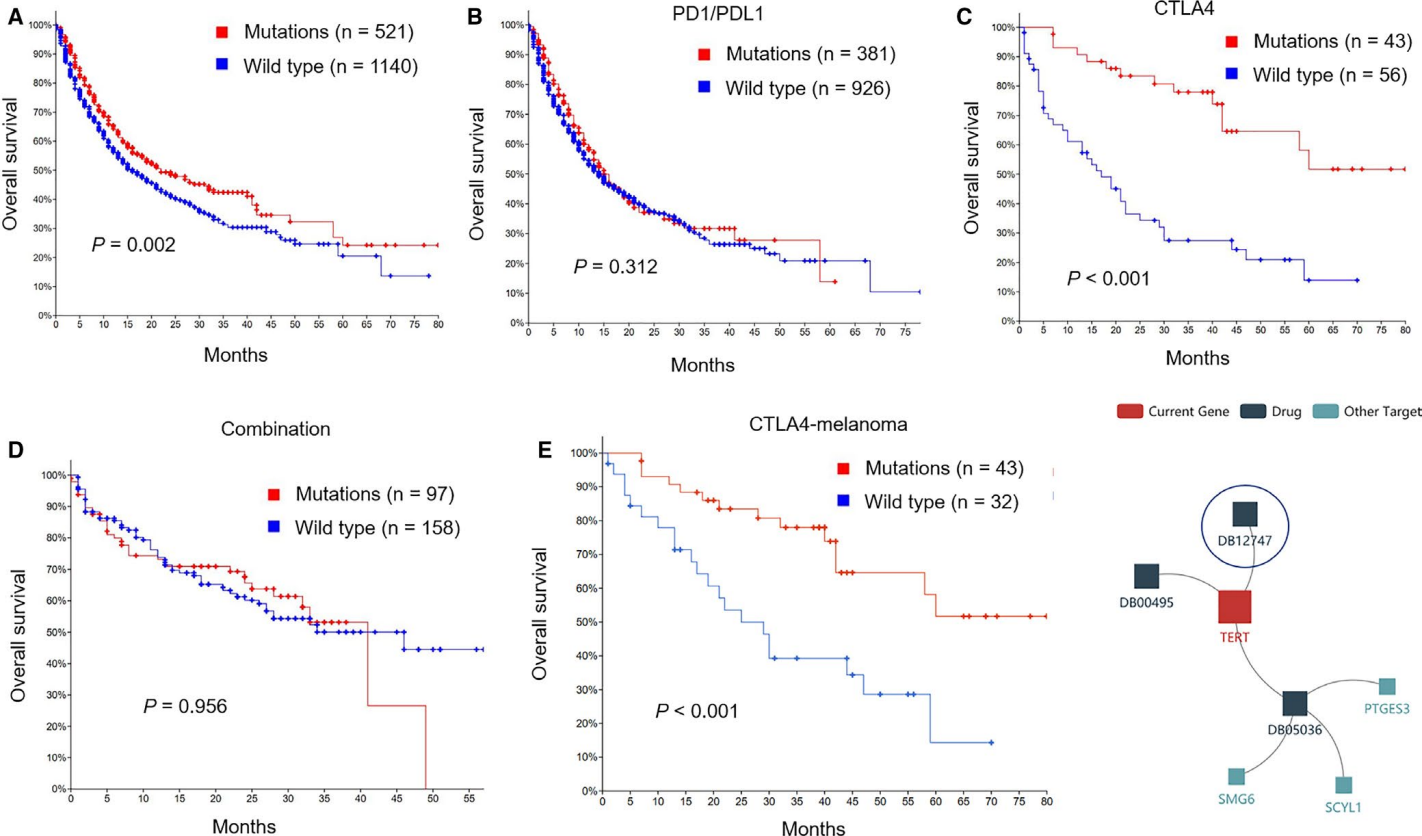
Overall survival of patients with *TERT* mutations vs wild type. A, Overall survival of patients with *TERT* mutations vs wild type in *ICI* treatment cohort (22.00 mo vs 16.00 mo, *P* = .002). B‐D, Overall survival of patients with *TERT* mutations vs wild type in monotherapy and combination treatment cohort (only a significant difference in the *anti‐CTLA4* group, mutations = 43, wild type = 56, *NA* vs 17.00 mo, *P* < .001). The red curve represents the *TERT* mutation group, and the blue curve represents the *TERT* wild‐type group. E, Overall survival of patients with melanoma with *TERT* mutations vs wild type in *anti‐CTLA4* cohort. The red curve represents the *TERT* mutation group, and the blue curve represents the *TERT* wild group. F, Prediction of targeted *TERT* drugs in the *TISIDB* database. The red square represents the current gene. The black square represents predicted drugs. The light blue square represents other targets

From the abovementioned results, we found that patients with *TERT* mutations had a worse prognosis. However, in patients who received *ICIs*, what was the relationship between *TERT* mutation and *OS*? Surprisingly, patients with *TERT* mutations (n = 521) in the *ICI* treatment cohort showed a significantly longer median *OS* than the wild‐type population (n = 1140) (Figure 3A, 22.00 months vs 16.00 months, *P* = .002).

To understand whether patients with *TERT* mutations can benefit from monotherapy or combination treatment, we conducted subgroup analysis in the monotherapy and combination treatment groups. Notably, only in the *anti‐CTLA4* group, patients with *TERT* mutation (n = 43) had longer *OS* than those with wild type (n = 56) (Figure 3C, NA vs 17.00 months, *P* < .001). However, there were no statistical differences in the *PD1*/*PDL1* blockade and combination treatment groups (Figure 3B, *P* = .312; Figure 3D, *P* = .956). We also found 99 patients who received *anti‐CTLA4* treatment, including 75 patients with melanoma (43 with *TERT* mutations and 32 with *TERT* wild type), 20 patients with breast cancer (all with *TERT* wild type), and four patients with other cancer types (also *TERT* wild type). Therefore, we further explored the predictive value of *TERT* mutation on the efficacy of *anti‐CTLA4* treatment in the melanoma group. The results showed that patients with melanoma with *TERT* mutations (n = 43) had longer *OS* than those with wild type (n = 32) (Figure 3E, *P* < .001). Currently, drugs targeting *TERT* have been under investigation and development. For instance, DB12747, DB00495, and DB05036 have been developed (Figure 3F). DB12747 (tertomotide) is an immunotherapy drug with mere targeting of *TERT*.

## 4 DISCUSSION

In our study, we first demonstrated a specific cancer spectrum of *TERT* mutation and *CNA* change in 10 336 patients or 10 945 samples to date. Strikingly, *TERT* mutation was common and found to be frequent in many malignancies, such as sex cord‐stromal tumor, bladder cancer, and glioma. Other surveys on different tumor types confirmed the high prevalence of *TERT* mutations in bladder cancer, and glioma, although they developed with different frequencies.^38^, ^39^, ^40^, ^41^ However, *TERT* mutation is not universal and has been absent, or rarely observed, in some cancer types such as breast cancer, prostate cancer, thymic tumor, histiocytosis, and mature *T* and *NK* neoplasms. Consistent results have also been obtained in breast cancer and prostate cancer with a lower mutation frequency.^38^, ^42^ In addition, we found that *TERT* co‐mutated with most genes. Therefore, we speculated that *TERT* mutation might play a role in the majority of cancers.

Tumor mutational burden, as a biomarker of response to *ICIs*, is closely related to the number of gene mutations and neoantigens.^13^, ^14^ Our results demonstrated that *TERT* mutation was ranked second in the top three mutation frequencies. Moreover, we mainly explored the correlation between *TERT* mutation and *TMB* score or neoantigens load in multiple cancer types. *TERT* mutation was significantly associated with high *TMB* score and neoantigen load in all cancer types. Tumor heterogeneity is the key to determining the tumor's immune response, and tumors with high heterogeneity can suppress the immune response.^43^ Our results showed that the tumor purity of the *TERT* mutation group was higher, indicating lower tumor heterogeneity. These findings highlight that *TERT* mutation may be related to the tumor's immune response.

To maintain immortal characteristics, malignant tumor cells constantly induce *TERT* mutations to aberrantly upregulate *TERT* expression, and ultimately enable telomere maintenance, which is tightly regulated in normal somatic cells.^15^, ^16^, ^17^, ^44^ In the present study, we first measured *TERT* expression in different types of tumors using the *TIMER* database. We found that the expression level of *TERT* was generally higher in various tumors, than in the adjacent normal tissue, which is consistent with previous studies. Therefore, *TERT* is frequently activated in many malignant tumors and closely related to cancer progression.

The *TME* comprises immune cells, mesenchymal cells, endothelial cells, and inflammatory mediators.^45^, ^46^ Immunocompetence, to some extent, partially reflects the microenvironment in which the tumor is involved. Previous studies have also provided an elegant analysis on how the activation of tumor‐intrinsic genes shapes *TME*.^47^, ^48^, ^49^ We first analyzed the correlation between *TERT* mutation and immune‐expression signatures. Lymphocyte infiltration, macrophage regulation, *IFN‐γ* response, and *TGF‐β* response which were representative immune‐expression signatures, all had higher gene signature scores in the *TERT* mutation group. Lymphocytes are cells characterized by high telomerase activity to maintain telomere length.^50^ Interestingly, the signature score of macrophage regulation, *IFN‐γ* response, and *TGF‐β* response in the *TERT* mutation group was also relatively higher. It is all known that both tumor‐associated macrophages and *TGF‐β* promote key processes in immunosuppression via effects on the *TME*.^51^, ^52^ *IFN‐γ* can also induce *M2* macrophage differentiation, which plays a suppressive role in immune function.^53^ We speculated that the *TERT* mutation may cause an immunosuppressive *TME*.

The infiltrating immune cell, an integral component of *TME*, is usually a heterogeneous mixture of immune cells, including cell types associated with activity and inhibition.^45^, ^54^ We further analyzed the relationship between *TERT* mutations and immune cells. Among the *Th* series cells, including *Th1*, *Th2*, and *Th17* cells, we found that the immune cell score of the *TERT* mutation group was lower. Similarly, the immune cell score of memory B cells in the *TERT* mutation group was also lower, while the immune cell score of naïve B cells was higher. Subsequently, we analyzed the distribution of T series cells in the *TERT* mutation group. Only the immune score of activated memory *CD4* memory T cell in the *TERT* mutation group was higher, and there was a significant difference. Subsequently, we also found significant differences in the nine types of immune cells. Activated dendritic cells, *M0* macrophages, *M1* macrophages, neutrophils, resting *NK* cells, and plasma cells had relatively higher immune scores in the *TERT* mutation group, while resting mast cells, monocytes, and activated *NK* cells had lower immune scores. The abovementioned results suggested that *TERT* mutation might play an important role in immunologic dysfunction and unique *TME*. Moreover, we also found that *TERT* mutation was related to worse prognosis in all cancer types. However, in patients who received *ICIs*, what was the relationship between *TERT* mutations and OS?

Finally, we investigated the relationship between *TERT* mutation and *ICIs*. Surprisingly, patients with *TERT* mutations in the *ICI* treatment cohort showed a significantly longer *OS* than the wild‐type population. Notably, in the subgroup analysis of monotherapy and combination treatment, only in the *anti‐CTLA4* group, patients with *TERT* mutations had a better prognosis. We further explored the predictive value of *TERT* mutation on the efficacy of *anti‐CTLA4* treatment in certain cancer types. However, the results showed that only patients with melanoma with *TERT* mutation could more likely benefit from *anti‐CTLA4* treatment. Therefore, *TERT* mutant patients may benefit from *anti‐CTLA4* treatment, especially for melanoma.

Our study may have some clinical relevance. Immunotherapeutic approaches targeting *TERT* have been evaluated in many clinical trials. For instance, tertomotide, a peptide vaccine that can activate the immune system to kill cancer cells, is under investigation in a clinical trial *NCT01223209*.^55^ Further immunologic targeting of *TERT* may represent a promising new aspect in cancer treatment.

This study also has certain limitations. First, our study was only a bioinformatic and pan‐cancer analysis of *anti‐CTLA4* treatment. The next step is to confirm whether *TERT* mutation is an immune predictive marker for a specific tumor or certain types of tumors through prospective or retrospective studies. Second, there were few studies on *anti‐CTLA4* treatment and no other data on *anti‐CTLA4* treatment were currently collected. We cannot use another cohort to verify our findings.

In conclusion, we have identified that *TERT* mutations are unevenly distributed in different cancer types which may lead to aberrant upregulation of *TERT* expression in various tumors. *TERT* mutations were significantly associated with higher *TMB* value and neoantigen load and may lead to an immunosuppressive microenvironment. *TERT* mutation was related to worse prognosis in the cBioPortal database and better prognosis in the *anti‐CTLA4* treatment cohort. Therefore, our study confirmed for the first time that *TERT* mutation could be used as a predictive marker for *anti‐CTLA4* treatment, especially for melanoma. Based on these data, further clinical trials are necessary to confirm whether *TERT* mutation, which is a potential predictor for *anti‐CTLA4* treatment and *TERT‐targeted* therapy combined with immunotherapy, has better benefits for *TERT* mutant patients.

## CONFLICT OF INTEREST

All authors declare that there is no conflict of interest.

## AUTHORS' CONTRIBUTIONS

Haiyong Wang designed the project and proposed the idea; Huahua Li wrote the manuscript; Jia Li carried out data download and literature collection; Chenyue Zhang conducted bioinformatics analysis; Chenxing Zhang conducted chart and statistical processing.

## ETHICAL APPROVAL

This study was approved by the Ethics Committee of the Shandong Cancer Hospital.

## CONSENT FOR PUBLICATION

All authors agree to publish.

## Data Availability

We declared that materials described in the manuscript, including all relevant raw data, will be freely available to any scientist wishing to use them for non‐commercial purposes, without breaching participant confidentiality.
